# A comparative study of anticoagulation combined with different catheter-directed thrombolysis strategies (urokinase catheter-directed vs. alteplase infusion) in the treatment of intermediate-risk pulmonary embolism

**DOI:** 10.3389/fcvm.2025.1675768

**Published:** 2026-01-02

**Authors:** Jinqi Huang, Qihong Chen

**Affiliations:** Department of Interventional Vascular Surgery, The First Hospital of Putian City, Putian, Fujian, China

**Keywords:** acute pulmonary embolism, alteplase, catheter-directed thrombolysis, efficacy comparison, intermediate-risk, urokinase

## Abstract

**Objective:**

To compare the clinical efficacy and safety of anticoagulation combined with catheter-directed urokinase (UK) thrombolysis vs. anticoagulation combined with catheter-directed alteplase (rt-PA) infusion thrombolysis in patients with intermediate-risk acute pulmonary embolism (APE).

**Methods:**

A retrospective analysis was conducted on intermediate-risk APE patients treated at our center between June 2022 and May 2025, all of whom received anticoagulation combined with catheter-directed thrombolysis. The UK group (*n* = 56) received continuous UK infusion via pulmonary artery catheter (400,000–500,000 IU/day for 2–3 days), while the rt-PA group (*n* = 23) received intraprocedural rt-PA infusion (20 mg/30 min). Changes in cardiac troponin I (cTnI), N-terminal pro-brain natriuretic peptide (NT-proBNP), right ventricular diameter/left ventricular diameter (RVD/LVD), and pulmonary artery obstruction index (PAOI) were compared before and after treatment.

**Results:**

Baseline characteristics showed no significant differences between groups (*P* > 0.05). Post-treatment, the UK group demonstrated significant improvements in PAOI, RVD/LVD, NT-proBNP, and cTnI (*P* < 0.05), while the rt-PA group showed significant improvements in PAOI, RVD/LVD, and cTnI (*P* < 0.05) but not NT-proBNP (*P* = 0.088). There were no statistically significant differences in the aforementioned indicators between the two groups at both pre-treatment and post-treatment time points (*P* > 0.05). There were no statistically significant differences in in-hospital mortality, the incidence of bleeding adverse events, and hospital stay duration between the two groups (*P* > 0.05).

**Conclusion:**

For intermediate-risk APE, catheter-directed UK thrombolysis and intraprocedural rt-PA infusion offer comparable efficacy and safety. However, the rt-PA regimen may be more advantageous due to its avoidance of prolonged catheter placement.

## Introduction

Acute pulmonary embolism (APE), a leading cause of cardiovascular mortality, requires careful selection of treatment strategies as they directly impact patient outcomes (1–3). While intermediate-risk APE patients do not meet high-risk criteria, the presence of right ventricular (RV) dysfunction confers a substantial risk of mortality in this population (4). For such patients, although systemic thrombolysis can reduce the risk of hemodynamic decompensation, it is not recommended for routine use due to its higher bleeding risk and lack of demonstrated overall mortality benefit (5). Current guidelines suggest considering catheter-directed thrombolysis (CDT) for this population to mitigate the bleeding risks associated with systemic thrombolysis (1, 6). Network meta-analyses indicate that CDT significantly reduces major bleeding and intracranial hemorrhage compared to systemic thrombolysis, while also demonstrating lower mortality risk (7). Additional meta-analyses confirm that CDT decreases all-cause and gastrointestinal bleeding without significantly increasing intracranial hemorrhage risk (8).

Despite these advances, the optimal dosing and infusion duration for locally administered thrombolytic agents—specifically urokinase (UK) and alteplase (rt-PA)—remain uncertain in APE management. It is noteworthy that significant variations exist in the CDT protocols (including medication type, dosage, delivery devices, and treatment duration) adopted across different clinical trials. For instance, completed trials such as SEATTLE II, CANARY, and others, along with the ongoing HI-PEITHO trial, have employed distinct rt-PA infusion protocols and durations (9–12). Our institution previously adopted a UK-CDT protocol (400,000–500,000 IU/day for 2–3 days) but recently transitioned to an intraprocedural rt-PA infusion regimen (20 mg/30 min). This fixed-dose short-infusion protocol references explorations of low-dose, short-duration rt-PA infusion regimens in trials such as OPTALYSE PE (13), aiming to evaluate whether it can maintain thrombolytic efficacy while avoiding risks associated with prolonged catheter indwelling. While the rt-PA protocol offers notable procedural advantages in terms of convenience, its clinical efficacy relative to UK-CDT requires further validation. To date, no studies have directly compared UK-CDT with intraprocedural rt-PA infusion for the treatment of intermediate-risk APE.

Against this background, this retrospective study compares the efficacy and safety of anticoagulation combined with UK-CDT vs. anticoagulation plus intraprocedural rt-PA infusion in intermediate-risk APE patients. The findings aim to provide critical evidence for optimizing thrombolytic agent selection and delivery protocols in CDT.

## Materials and methods

### Patient selection and grouping

We retrospectively enrolled patients with intermediate-risk APE who underwent anticoagulation combined with CDT at our center between June 2022 and May 2025. Inclusion criteria: (1) Intermediate-risk patients (with imaging evidence of RV dysfunction or elevated cardiac biomarkers); (2) Time from symptom onset to treatment ≤14 days; (3) Underwent either UK-CDT (UK group) or catheter-directed intraprocedural rt-PA thrombolysis (rt-PA group). Exclusion criteria: (1) High-risk patients (with hypotension or shock); (2) Low-risk patients (without imaging evidence of RV dysfunction or elevated cardiac biomarkers); (3) Subacute or chronic pulmonary embolism (time from symptom onset to treatment >14 days); (4) Underwent percutaneous mechanical thrombectomy. The CDT protocols used in this study adhered to the following major contraindications: (1) active bleeding or high risk of bleeding; (2) recent (within 3 months) intracranial or spinal surgery/trauma; (3) known allergy to the thrombolytic agents used. During the study period, none of the intermediate-risk APE patients included in the analysis presented with these contraindications to CDT.

### Treatment protocols

Anticoagulation therapy: During CDT, patients in the UK group were administered intravenous unfractionated heparin to maintain an activated partial thromboplastin time at 1.5–2.0 times the normal value. For patients in the UK group during periods not receiving CDT, as well as for patients in the rt-PA group, subcutaneous injection of low-molecular-weight heparin was used, with a dosage of 100 IU/kg body weight administered every 12 h.

Interventional therapy: For patients concurrently diagnosed with acute lower extremity deep vein thrombosis, a retrievable inferior vena cava filter was placed prior to thrombolysis. A 5 F Pigtail catheter was positioned in the pulmonary artery via one femoral vein approach. In both groups, CDT was achieved with a unilateral catheterization strategy utilizing the TEMPO™ diagnostic catheter (Cordis, USA).

UK group: The catheter was selectively placed in one of the pulmonary arteries, typically the one with the greater thrombus burden, and UK was continuously infused through the Pigtail catheter at a dose of 400,000–500,000 IU per day. Pulmonary angiography was repeated every 1–2 days, and the position of the Pigtail catheter was adjusted as needed. The duration of thrombolysis was typically controlled within 2–3 days. The decision to terminate CDT was guided by a comprehensive assessment of clinical improvement and evidence of thrombus resolution on follow-up angiography.

rt-PA group: The Pigtail catheter was positioned in the main pulmonary artery, and rt-PA was pumped in through the catheter (total dose of 20 mg), with the infusion set to be completed within 30 min.

All CDT procedures were performed by the same team of interventional vascular surgeons at our center. This team consisted of three primary operators, each with over 10 years of experience in vascular interventional diagnosis and treatment. During the study period, our center performed approximately 30–40 pulmonary embolism-related interventional procedures annually (including CDT and mechanical thrombectomy). This volume ensures that the operator team possesses stable proficiency and extensive clinical experience in managing intermediate-risk APE. Furthermore, the treatment strategy for patients with intermediate-risk APE at our center was established through multidisciplinary consultation involving experts from interventional vascular surgery, cardiology, and respiratory and critical care medicine, following a workflow analogous to that of a Pulmonary Embolism Response Team (PERT).

### Outcome measures and definitions

The data on the pre-treatment pulmonary artery obstruction index (PAOI), right ventricular diameter/left ventricular diameter (RVD/LVD), N-terminal pro-brain natriuretic peptide (NT-proBNP), and cardiac troponin I (cTnI) represent the values measured from the time of patient admission until immediately before the intervention. In contrast, the post-treatment data for these indicators represent the values measured from the completion of the intervention until the time of discharge.

PAOI and RVD/LVD quantification: In pulmonary artery computed tomography angiography, the measurement method for RVD/LVD and the calculation method for Mastora PAOI can be referred to in the relevant literature by Gao et al (14). To minimize subjectivity, all assessments were independently performed by a vascular surgeon with 7 years of experience who was blinded to patient grouping.

Biomarker thresholds: cTnI positivity: >0.03 μg/L (institutional upper reference limit). NT-proBNP positivity: The upper limit of normal for NT-proBNP varies with age: it is 450 ng/L for individuals under 50 years old, 900 ng/L for those aged between 50 and 75 years, and 1800 ng/L for individuals over 75 years old.

Bleeding events were classified according to the GUSTO criteria: severe bleeding was defined as intracranial hemorrhage, hemodynamic instability requiring intervention, a hemoglobin drop of ≥5 g/dL, or the need for transfusion of ≥4 units of packed red blood cells. Moderate bleeding included overt bleeding (e.g., gastrointestinal bleeding) or a hemoglobin drop of ≥3 g/dL but <5 g/dL, or the need for blood transfusion without meeting the criteria for severe bleeding. Minor bleeding referred to other bleeding events that did not meet the above criteria and did not require intervention.

### Statistical analysis

Statistical analyses were performed using SPSS 23.0, with categorical variables presented as counts (percentages) and compared using chi-square tests, while non-normally distributed continuous variables were expressed as medians (first quartile, third quartile) and analyzed with Mann–Whitney U tests for intergroup comparisons and Wilcoxon signed-rank tests for intragroup pre-post treatment comparisons, with statistical significance set at *P* < 0.05. Multiple comparisons were present in this study. As an exploratory analysis, *p*-values were not adjusted to reduce the risk of false-negative results, which should be considered when interpreting the findings.

To minimize confounding effects, propensity score matching (PSM) was conducted using 1:1 nearest-neighbor matching (caliper = 0.25) with matching variables including gender, age, syncope, NT-proBNP positivity, cTnI positivity, baseline PAOI, and baseline RVD/LVD, resulting in 19 matched pairs (19 patients each in UK and rt-PA groups) after analysis performed using R programming language, along with Zstats v1.0 (http://www.zstats.net).

## Results

### Comparison of baseline characteristics between the two groups

There were no statistically significant differences in baseline characteristics between the two groups (*P* > 0.05) (Table 1.)

**Table 1:** Comparison of baseline characteristics between the Two groups of patients.

Parameters	UK group (*n* = 56)	rt-PA group (*n* = 23)	*χ*^2^/Z-value	*P*-value
Gender				
Male (*n* = 26)	17 (30.36%)	9 (39.13%)	0.568	0.451
Female (*n* = 53)	39 (69.64%)	14 (60.87%)		
Age (years)	65 (59, 71)	66 (59, 73)	−0.583	0.560
Time from symptom onset to treatment (days)	3 (1, 7)	3 (1, 7)	−0.500	0.617
Deep vein thrombosis of lower extremities				
None (*n* = 15)	11 (19.64%)	4 (17.39%)	0.213	0.975
Left side (*n* = 19)	14 (25.00%)	5 (21.74%)		
Right side (*n* = 23)	16 (28.57%)	7 (30.43%)		
Bilateral (*n* = 22)	15 (26.79%)	7 (30.43%)		
Syncope				
None (*n* = 59)	39 (69.64%)	20 (86.96%)	2.585	0.108
Present (*n* = 20)	17 (30.36%)	3 (13.04%)		
Hypertension				
None (*n* = 41)	29 (51.79%)	12 (52.17%)	0.001	0.975
Present (*n* = 38)	27 (48.21%)	11 (47.83%)		
Diabetes mellitus				
None (*n* = 67)	47 (83.93%)	20 (86.96%)	<0.001	1.000
Present (*n* = 12)	9 (16.07%)	3 (13.04%)		
Coronary heart disease				
None (*n* = 69)	49 (87.50%)	20 (86.96%)	<0.001	1.000
Present (*n* = 10)	7 (12.50%)	3 (13.04%)		
Hyperlipidemia				
None (*n* = 27)	20 (35.71%)	7 (30.43%)	0.202	0.653
Present (*n* = 52)	36 (64.29%)	16 (69.57%)		
History of venous thromboembolism				
None (*n* = 67)	48 (85.71%)	19 (82.61%)	<0.001	0.997
Present (*n* = 12)	8 (14.29%)	4 (17.39%)		
NT-proBNP				
Negative (*n* = 29)	20 (46.51%, *n* = 43)	9 (39.13%)	0.331	0.565
Positive (*n* = 37)	23 (53.49%, *n* = 43)	14 (60.87%)		
cTnI				
Negative (*n* = 26)	18 (39.13%, *n* = 46)	8 (34.78%)	0.123	0.725
Positive (*n* = 43)	28 (60.87%, *n* = 46)	15 (65.22%)		
Inferior vena cava filter placement				
None (*n* = 14)	9 (16.07%)	5 (21.74%)	0.076	0.783
Present (*n* = 65)	47 (83.93%)	18 (78.26%)		

Continuous variables are presented as median (interquartile range); Data on NT-proBNP and cTnI are missing for some patients.

### Efficacy results

After treatment, the UK group showed significant improvements in PAOI, RVD/LVD, NT-proBNP, and cTnI compared to the pre-treatment values (*P* < 0.05). In the rt-PA group, significant improvements were also observed in PAOI, RVD/LVD, and cTnI (*P* < 0.05), except for NT-proBNP (*P* = 0.088). No statistically significant differences were found between the two groups in the aforementioned indicators at either the pre-treatment or post-treatment time points (*P* > 0.05) (Tables 2, 3). The standardized mean differences for all matched variables were < 0.23. The statistical analysis tables before and after matching are shown in Supplementary Tables S1 and S2. After matching, there were still no statistically significant differences in post-treatment PAOI, RVD/LVD, NT-proBNP, and cTnI between the two groups (*P* > 0.05) (Table 4).

**Table 2:** Comparison of PAOI, RVD/LVD, NT-proBNP, and cTnI before and after treatment between groups.

Group/parameter	*n*	Pre-treatment PAOI (%)	*n*	Post-treatment PAOI (%)
UK group	53	45.16 (35.49, 56.45)	46	20.00 (8.71, 25.17)
rt-PA group	23	47.74 (33.55, 56.13)	18	15.81 (9.19, 20.81)
*Z-*value		−0.254		−1.128
*P-*value		0.799		0.259
		Pre-treatment RVD/LVD		Post-treatment RVD/LVD
UK Group	53	1.38 (1.07, 1.63)	46	1.09 (1.02, 1.35)
rt-PA Group	23	1.51 (1.19, 1.74)	18	1.09 (0.98, 1.27)
*Z-*value		−1.306		−0.635
*P-*value		0.192		0.525
		Pre-treatment NT-proBNP (ng/L)		Post-treatment NT-proBNP (ng/L)
UK group	43	958.78 (403.00, 2,302.34)	33	84.00 (52.63, 229.48)
rt-PA group	23	1,147.00 (213.20, 2,204.00)	15	176.00 (66.72, 606.11)
*Z-*value		−0.074		−1.235
*P-*value		0.941		0.217
		Pre-treatment cTnI (*μ*g/L)		Post-treatment cTnI (μg/L)
UK group	46	0.08 (<0.01, 0.15)	32	0.01 (0.01, 0.02)
rt-PA group	23	0.11 (<0.01, 0.31)	14	0.02 (0.01, 0.05)
*Z-*value		−0.459		−1.087
*P-*value		0.646		0.277

Continuous variables are presented as median (interquartile range); Data is missing for some patients.

**Table 3:** Comparison of PAOI, RVD/LVD, NT-proBNP, and cTnI before and after treatment within each group.

Group	*n*	Pre-treatment PAOI (%)	Post-treatment PAOI (%)	*Z-*value	*P-*value
UK group	44	45.81 (38.22, 57.26)	19.03 (8.06, 24.36)	−5.778	<0.001
rt-PA group	18	48.07 (35.00, 56.78)	15.81 (9.19, 20.81)	−3.680	<0.001
		Pre-treatment RVD/LVD	Post-treatment RVD/LVD		
UK group	44	1.39 (1.08, 1.62)	1.10 (1.03, 1.37)	−2.597	0.009
rt-PA group	18	1.50 (1.19, 1.74)	1.09 (0.98, 1.27)	−2.593	0.010
		Pre-treatment NT-proBNP (ng/L)	Post-treatment NT-proBNP (ng/L)		
UK group	25	944.00 (329.85, 1,983.34)	77.00 (45.13, 211.07)	−4.076	<0.001
rt-PA group	15	1,234.00 (198.30, 3,965.00)	176.00 (66.72, 606.11)	−1.704	0.088
		Pre-treatment cTnI (μg/L)	Post-treatment cTnI (μg/L)		
UK group	30	0.08 (0.01, 0.28)	0.01 (<0.01, 0.02)	−4.247	<0.001
rt-PA group	14	0.12 (0.03, 0.31)	0.02 (0.01, 0.05)	−2.417	0.016

Continuous variables are presented as median (interquartile range); Data is missing for some patients.

**Table 4:** Comparison of efficacy indicators between the Two groups of patients after PSM.

Efficacy indicator	UK group (*n* = 19)	rt-PA group (*n* = 19)	*Z*-value	*P*-value
Post-treatment PAOI (%)	21.94 (12.90, 34.52) (*n* = 13)	15.48 (10.32, 20.65) (*n* = 15)	−1.636	0.102
Post-treatment RVD/LVD	1.08 (0.99, 1.51) (*n* = 13)	1.11 (0.99, 1.31) (*n* = 15)	−0.046	0.963
Post-treatment NT-proBNP (ng/L)	127.62 (59.46, 2,754.10) (*n* = 12)	278.00 (79.50, 612.42) (*n* = 12)	−0.577	0.564
Post-treatment cTnI (μg/L)	0.02 (<0.01, 0.04) (*n* = 10)	0.02 (0.01, 0.04) (*n* = 12)	−0.594	0.553

Continuous variables are presented as median (interquartile range); Data is missing for some patients.

### Safety results

No statistically significant differences were observed between the two groups in terms of in-hospital mortality, the incidence of hemorrhagic adverse events, or the length of hospital stay (*P* > 0.05). The median CDT duration in the UK group was 2 days (interquartile range: 2–3 days). A total of three hemorrhagic events were recorded in the UK group: one case of right frontal lobe hemorrhage (classified as severe bleeding) occurred 2 days post-intervention, and the patient unfortunately passed away 7 days post-intervention; another case of right renal hemorrhage (also classified as severe bleeding) occurred 1 day post-intervention, with the patient succumbing to shock and cardiopulmonary failure 4 days post-intervention; and one case of left rectus abdominis and pelvic hemorrhage (classified as moderate bleeding) occurred 3 days post-intervention. In the rt-PA group, one case of left eye subconjunctival hemorrhage (classified as minor bleeding) was recorded 1 day post-intervention (Table 5). Similarly, no significant differences were observed in in-hospital mortality, the incidence of hemorrhagic events, or the length of hospital stay between the two groups after matching (*P* > 0.05) (Table 6).

**Table 5:** Comparison of in-hospital mortality, bleeding adverse events, and length of hospital stay between the Two groups.

Safety indicator	UK group (*n* = 56)	rt-PA group (*n* = 23)	χ^2^/*Z*-value	*P*-value
In-hospital mortality (n, %)	2 (3.57%)	0	–	1.000^a^
Bleeding events (n, %)	3 (5.36%)	1 (4.35%)	<0.001	1.000^b^
Length of hospital stay (days)	6 (5, 9)	7 (5, 9)	−0.513	0.608

a Fisher's exact test was applied.

b Chi-square test with continuity correction was used; Continuous variables are presented as median (interquartile range).

**Table 6:** Comparison of safety indicators between the Two groups after PSM.

Safety indicator	UK group (*n* = 19)	rt-PA group (*n* = 19)	*Z*-value	*P*-value
In-hospital mortality (n, %)	1 (5.26%)	0	–	1.000^a^
Bleeding events (n, %)	1 (5.26%)	1 (5.26%)	–	1.000^a^
Length of hospital stay (days)	6 (5, 9)	7 (5, 9)	−0.413	0.679

a Fisher's exact test was used for statistical analysis; Continuous variables are presented as median (interquartile range).

## Discussion

This study compared the efficacy and safety of anticoagulation combined with different CDT strategies (UK-CDT vs. intraoperative rt-PA infusion) in the treatment of intermediate-risk APE. The study employed PAOI, RVD/LVD, NT-proBNP, and cTnI as evaluation indicators, reflecting thrombus burden, RV function impairment, and myocardial injury, respectively. Although both treatment regimens significantly improved thrombus burden, RV function, and myocardial injury markers, the reduction in NT-proBNP in the rt-PA group did not reach statistical significance (*P* = 0.088). This discrepancy may be related to the relatively small sample size in the rt-PA group and warrants further investigation for validation. Notably, there were no significant differences in the direct comparisons of all efficacy and safety indicators between the two groups, and this conclusion remained consistent after PSM, suggesting that the short-term efficacy of intraoperative rt-PA infusion is comparable to that of UK-CDT. Furthermore, although the two severe bleeding events (both fatal) in the UK group may be associated with prolonged catheterization, the sample size limitation necessitates larger-scale studies to confirm any differences in bleeding risk between the groups.

There is still controversy in current guidelines regarding whether the routine use of CDT is necessary for patients with intermediate-risk APE. However, several studies in recent years have suggested that CDT may confer significant benefits for this group of patients (15–17). For instance, Kroupa et al. (18) found that the improvement rate of pulmonary artery systolic pressure at 24 h after treatment was significantly higher in the CDT group compared to the anticoagulation-only group (*P* = 0.001). Sadeghipour et al. (10) demonstrated that the incidence of RV dysfunction (RVD/LVD > 0.9) at 72 h after treatment was significantly lower in the CDT group than in the anticoagulation group (27.0% vs. 52.1%, *P* = 0.01). Kabrhel et al. (19) conducted a cost-effectiveness analysis of three treatment strategies (anticoagulation alone, systemic thrombolysis, and CDT) for intermediate-risk APE patients. The results indicated that CDT provided the greatest quality-adjusted life-year benefits. Based on this evidence, our center routinely adopts a treatment regimen combining anticoagulation with CDT in clinical practice.

By comparing different rt-PA administration regimens, we found that despite significant differences in the mode of administration and infusion duration, the clinical efficacy was similar. Compared to the standard rt-PA regimen (24 mg total dose: unilateral catheter infusion at 1 mg/h for 24 h, or bilateral catheter infusion at 1 mg/h per catheter for 12 h) in the SEATTLE II trial (9), our intraoperative rapid infusion protocol (20 mg administered over 30 min) achieved comparable improvement in RV function (reduction in RVD/LVD ratio from 1.5 to 1.1) while significantly shortening the procedural time. Similarly, Kroupa et al. (18) employed a CDT protocol with a total rt-PA dose of 20 mg (bilateral catheter infusion at 1 mg/h per catheter for 10 h), and Akin et al. (20) conducted a study (submassive APE group: average rt-PA dose of 26 mg, infused over 10 min to 19 h). Although the infusion durations varied among these studies, their efficacy outcomes were comparable to those of our study, and all demonstrated favorable safety profiles. Collectively, these findings suggest that the clinical efficacy of rt-PA may primarily depend on the total administered dose (20–26 mg) rather than the infusion duration. Although the potential bleeding risk associated with rapid infusion protocols still requires validation in larger samples, the safety data from our study are encouraging (only one case of mild subconjunctival hemorrhage), providing important evidence for optimizing rt-PA administration regimens. The results of the OPTALYSE PE trial further support the exploration of optimal rt-PA dosing and catheterization duration, demonstrating that even an ultra-low-dose rt-PA CDT protocol (total dose of 4–12 mg per lung) combined with a shortened infusion time (2–6 h) can achieve significant clinical efficacy (13).

In this study, compared to intraoperative rt-PA infusion, UK-CDT treatment exhibited the following shortcomings: (1) It typically required a longer duration of catheter placement (21), increasing the risk of catheter-related thrombosis and infection (22–24); (2) Due to femoral vein puncture and catheterization, patients needed to remain bedridden for extended periods, leading to poor comfort; (3) It necessitated additional Digital Subtraction Angiography examinations to evaluate efficacy, which not only increased radiation exposure for both medical staff and patients but also incurred extra costs; (4) Frequent monitoring of coagulation function and complete blood counts was required during treatment. Based on these reasons, we switched to intraoperative rt-PA infusion therapy, which could avoid the aforementioned drawbacks of UK-CDT. Although the intraoperative thrombolysis time in the rt-PA group (30 min) was significantly shorter than the catheter-directed thrombolysis time in the UK group (2–3 days), the hospital stay duration was similar between the two groups. This is related to the current standardized postoperative management protocol, where patients in both groups are discharged only after a follow-up evaluation scheduled 3–5 days after the interventional procedure (counting from the day of the procedure). With future optimization of the rt-PA treatment protocol, it is expected that the hospital stay can be further shortened by scheduling earlier follow-up assessments.

Limitations of the study: As a single-center, retrospective, non-randomized controlled study with a limited sample size, the conclusions of this research should be interpreted with caution and require validation through future multi-center, prospective, large-sample, randomized controlled trials. Additionally, there is a lack of long-term follow-up data, such as information on RV function and the incidence of chronic thromboembolic pulmonary hypertension. It should be noted that all patients received standardized anticoagulation therapy as the foundation treatment, whose central role may have partially obscured potential differences between the two CDT strategies. It should be noted that the optimal management for intermediate-risk APE remains unclear. Several ongoing trials, such as HI-PEITHO (12), are currently investigating the role of CDT in the care of these patients. Since our study only compared two specific modalities of CDT, it does not address the broader question of where CDT fits into the overall treatment paradigm for intermediate-risk APE, particularly in centers where mechanical thrombectomy is also routinely available.

In conclusion, this study demonstrated that for patients with intermediate-risk APE, no statistically significant differences were observed between anticoagulation combined with UK-CDT and anticoagulation combined with intraoperative transcatheter rt-PA infusion in terms of primary efficacy outcomes (reduction in thrombus burden, improvement in RV function) and safety endpoints (incidence of bleeding events). However, it is noteworthy that the rt-PA infusion protocol, by avoiding prolonged catheter indwelling, showed significant advantages in clinical practice. The findings of this study are hypothesis-generating and warrant further validation of this novel rt-PA CDT regimen in large, multicenter, randomized controlled trials.

## Data Availability

The raw data supporting the conclusions of this article will be made available by the authors, without undue reservation.
