# Supplementary material for: A comparative study of anticoagulation combined with different catheter-directed thrombolysis strategies (urokinase catheter-directed vs. alteplase infusion) in the treatment of intermediate-risk pulmonary embolism
Source: Front Cardiovasc Med. 2026 Jan 2;12:1675768. doi: 10.3389/fcvm.2025.1675768 (PMC12808480; doi:10.3389/fcvm.2025.1675768)
Supplement: Supplementary file 1 [file Datasheet1.pdf]

Supplementary Table 1. Statistical Analysis Before Propensity Score Matching

Variable	Total (n = 79)	UK group (n = 56)	rt-PA group (n = 23)	Z/χ^2	*P*	SMD
Gender						
Male	26 (32.91%)	17 (30.36%)	9 (39.13%)	0.568	0.451	0.180
Female	53 (67.09%)	39 (69.64%)	14 (60.87%)			-0.180
Age (years)	65 (59, 71)	65 (59, 71)	66 (59, 73)	-0.583	0.560	0.119
Syncope						
None	59 (74.68%)	39 (69.64%)	20 (86.96%)	2.585	0.108	0.514
Present	20 (25.32%)	17 (30.36%)	3 (13.04%)			-0.514
NT-proBNP						
Negative	29 (43.94%) (n = 66)	20 (46.51%) (n = 43)	9 (39.13%)	0.331	0.565	-0.151
Positive	37 (56.06%) (n = 66)	23 (53.49%) (n = 43)	14 (60.87%)			0.151
cTnI						
Negative	26 (37.68%) (n = 69)	18 (39.13%) (n = 46)	8 (34.78%)	0.123	0.725	-0.091
Positive	43 (62.32%) (n = 69)	28 (60.87%) (n = 46)	15 (65.22%)			0.091
Pre-treatment PAOI (%)	45.81 (34.03, 56.13) (n = 76)	45.16 (35.49, 56.45) (n = 53)	47.74 (33.55, 56.13)	-0.254	0.799	0.081
Pre-treatment RVD/LVD	1.42 (1.10, 1.68) (n = 76)	1.38 (1.07, 1.63) (n = 53)	1.51 (1.19, 1.74)	-1.306	0.192	0.317

Note: Continuous variables are presented as median (interquartile range); SMD, Standardized Mean Difference; Data is missing for some patients.

Supplementary Table 2. Statistical Analysis After Propensity Score Matching

Variable	Total (n = 38)	UK group (n = 19)	rt-PA group (n = 19)	Z	*P*	SMD
Gender						
Male	14 (36.84%)	6 (31.58%)	8 (42.11%)	-	0.737*	0.213
Female	24 (63.16%)	13 (68.42%)	11 (57.89%)			-0.213
Age (years)	67 (58, 71)	67 (58, 71)	66 (58, 72)	-0.073	0.942	-0.033
Syncope						
None	32 (84.21%)	16 (84.21%)	16 (84.21%)	-	1.000*	0
Present	6 (15.79%)	3 (15.79%)	3 (15.79%)			0
NT-proBNP						
Negative	17 (44.74%)	8 (42.11%)	9 (47.37%)	-	1.000*	0.105
Positive	21 (55.26%)	11 (57.89%)	10 (52.63%)			-0.105
cTnI						
Negative	14 (36.84%)	8 (42.11%)	6 (31.58%)	-	0.737*	-0.226
Positive	24 (63.16%)	11 (57.89%)	13 (68.42%)			0.226
Pre-treatment PAOI (%)	42.26 (33.55, 54.51)	41.94 (38.71, 49.68)	43.87 (33.55, 56.13)	-0.321	0.748	0.026
Pre-treatment RVD/LVD	1.48 (1.18, 1.65)	1.49 (1.12, 1.65)	1.35 (1.18, 1.69)	-0.350	0.726	-0.158

Note: Continuous variables are presented as median (interquartile range); SMD, Standardized Mean Difference; *Fisher's exact test was used for statistical analysis.
